# Endoscopic Retrograde Cholangiopancreatography‐guided Biliary Drainage with Duckbill‐type Anti‐reflux Metal Stent versus Endoscopic Ultrasound‐guided Hepaticogastrostomy for Malignant Distal Biliary Obstruction in Pancreatic Cancer with Duodenal Invasion

**DOI:** 10.1002/deo2.70154

**Published:** 2025-06-06

**Authors:** Tsuyoshi Takeda, Takashi Sasaki, Tatsuki Hirai, Yoichiro Sato, Yuri Maegawa, Takafumi Mie, Takaaki Furukawa, Yukari Suzuki, Takeshi Okamoto, Masato Ozaka, Naoki Sasahira

**Affiliations:** ^1^ Department of Hepato‐Biliary‐Pancreatic Medicine Cancer Institute Hospital of Japanese Foundation for Cancer Research Tokyo Japan

**Keywords:** anti‐reflux metal stent, duodenal invasion, endoscopic retrograde cholangiopancreatography, endoscopic ultrasound‐guided hepaticogastrostomy, malignant distal biliary obstruction

## Abstract

**Background:**

Duodenal invasion is a risk factor for early recurrent biliary obstruction (RBO) due to the increased risk of duodenobiliary reflux. Transpapillary biliary drainage using anti‐reflux metal stents (ARMS) and endoscopic ultrasound‐guided hepaticogastrostomy (EUS‐HGS) are two different strategies for this condition.

**Methods:**

We retrospectively reviewed unresectable pancreatic cancer (PC) patients with duodenal invasion who underwent either transpapillary biliary drainage using duckbill‐type ARMS (D‐ARMS) or EUS‐HGS for malignant distal biliary obstruction (MDBO). Technical and clinical success, causes of RBO, non‐RBO adverse events (AEs), time to RBO (TRBO), and endoscopic reintervention (ERI) were compared between groups.

**Results:**

Forty‐four patients were included (D‐ARMS: 22, EUS‐HGS: 22). Technical and clinical success rates, and non‐RBO AE rates (9.1% vs. 36.4%, *p* = 0.069) were not significantly different between groups. Common causes of RBO were biliary debris/stones in the D‐ARMS group and hyperplasia in the EUS‐HGS group. Overall RBO rates (33.3% vs. 45.0%, *p* = 0.53), median TRBO (246 vs. 222 days, *p* = 0.98), and outcomes after ERI were comparable between groups.

**Conclusions:**

Transpapillary biliary drainage using D‐ARMS may be a viable option in managing MDBO with duodenal invasion, especially for non‐high‐volume centers, when both procedures are technically feasible.

## 1 Introduction

Endoscopic placement of self‐expandable metal stents (SEMSs) is the gold standard for managing malignant distal biliary obstruction (MDBO) in recent guidelines [1, 2]. However, patients with duodenal invasion or a concomitant duodenal stent are prone to early recurrent biliary obstruction (RBO) because of the high risk of duodenal biliary reflux [3, 4, 5]. To mitigate the risk of early RBO caused by duodenobiliary reflux, transpapillary SEMS placement using anti‐reflux metal stents (ARMS) [6, 7, 8] and endoscopic ultrasound‐guided biliary drainage (EUS‐BD) [9, 10, 11] are new options that may extend stent patency in this condition.

With respect to EUS‐BD, as duodenobiliary reflux remains a major issue with EUS‐guided choledochoduodenostomy using conventional SEMS, EUS‐guided hepaticogastrostomy (EUS‐HGS) has been increasingly performed, with a previous study reporting longer time to RBO (TRBO) and lower adverse event (AE) rate in those undergoing EUS‐HGS [12]. EUS‐HGS can be performed even when transpapillary biliary drainage is technically unfeasible because of duodenal invasion, while it may be technically difficult when the bile duct is not dilated or the patient has severe ascites. On the other hand, endoscopic retrograde cholangiopancreatography (ERCP)‐guided biliary drainage using ARMS can be easily performed in such circumstances, when transpapillary biliary drainage is technically feasible. To date, no studies have directly compared ERCP‐guided biliary drainage using ARMS and EUS‐HGS in MDBO with duodenal invasion, where transpapillary biliary drainage is technically feasible.

Therefore, this study examined the efficacy and safety of ERCP‐guided biliary drainage using ARMS compared to EUS‐HGS in unresectable pancreatic cancer (PC) patients with duodenal invasion.

## 2 Methods

### 2.1 Patients

From our prospectively maintained database, we retrospectively reviewed unresectable PC patients with duodenal invasion who underwent either ERCP‐guided biliary drainage using duckbill‐type ARMS (D‐ARMS) or EUS‐HGS for MDBO at our hospital from February 2016 until March 2024. We excluded the following patients: 1) those with massive ascites precluding EUS‐HGS, 2) those where transpapillary biliary drainage was not technically feasible, or those with a history of post‐ERCP pancreatitis precluding ERCP‐guided biliary drainage. ERCP‐guided biliary drainage using D‐ARMS or EUS‐HGS was primarily selected depending on the period the procedure was performed, with a preference for EUS‐HGS after 2022. This was partly because we expanded the indications for EUS‐HGS in line with our institution's learning curve, shifting to perform EUS‐HGS at an earlier time period (e.g. before duodenal obstruction becomes a problem). This study was approved by the ethics committee of our institution (2023‐GB‐077) and performed according to the Declaration of Helsinki. Written informed consent for this study was waived by the ethics committee of our institution due to its retrospective design.

### 2.2 ERCP and EUS‐HGS

ERCP was conducted using a duodenoscope (JF260, TJF260, and TJF‐Q290V; Olympus Medical Systems, Tokyo, Japan) under conscious sedation. The ARMS used in this study was a fully covered laser‐cut type SEMS with a 12.5 mm duckbill‐shaped anti‐reflux valve attached to the distal end (Kawasumi Duckbill Biliary Stent, Kawasumi Laboratories Inc., Tokyo, Japan). All stents used were 10 mm in diameter and 6 or 8 cm in length. Stent length was determined according to cholangiographic findings, and the anti‐reflux valve was positioned below the papilla. The decision to place a prophylactic pancreatic stent or administer rectal nonsteroidal anti‐inflammatory drugs (NSAIDs) was left to the treating endoscopist's discretion.

EUS‐HGS was conducted using a convex echoendoscope (GF‐UCT260; Olympus Medical Systems, Tokyo, Japan) under conscious sedation. The SEMS used in this study was a partially covered SEMS (Niti‐S S‐type Stent, Taewoong Medical Co., Gimpo, Korea; EGIS Biliary Stent, Double Covered, S&G Biotech Inc., Yongin, Korea; Niti‐S Spring Stopper Stent, Taewoong Medical Co., Gimpo, Korea), which was deployed between the left intrahepatic duct (generally B3) and the stomach. All stents used were 8 or 10 mm in diameter and 8, 10, or 12 cm in length. For those who failed to insert an SEMS (*n* = 2), a dedicated 7Fr plastic stent was used (Through&Pass TYPE IT; Gadelius Medical, Tokyo, Japan).

### 2.3 Outcome Measures

Clinical outcomes were evaluated using Tokyo Criteria 2024 [13]. The primary outcome was TRBO, defined as the time from stent placement to biliary drainage or stent removal for RBO. The secondary outcomes included technical success, clinical success, causes of RBO, adverse events (AEs), overall survival (OS), and endoscopic reintervention (ERI). Technical success was defined as successful SEMS placement at the intended location. Patients who failed to place an SEMS were treated as technical failure. Clinical success was defined as a 50% decrease or normalization of total bilirubin within 14 days, for those who achieved technical success. RBO was defined as stent occlusion or stent migration requiring biliary drainage or stent removal, which was evaluated only in those who achieved technical and clinical successes. AEs were defined as any event requiring treatment in addition to RBO and were reported as non‐RBO and RBO AEs. AEs were categorized into early (within 14 days) and late (after 15 days), and their severity was graded using the American Society of Gastrointestinal Endoscopy lexicon guidelines [14]. Non‐occlusion cholangitis was dealt with as RBO when endoscopic biliary drainage was required, while it was dealt with as non‐RBO AEs when it was managed conservatively without requiring any endoscopic biliary drainage. Transient postprocedural fever, self‐limiting pain, asymptomatic pneumoperitoneum, and asymptomatic elevation of inflammatory markers, which are often observed after EUS‐HGS, were not dealt with as non‐RBO AEs. OS was defined as the time from stent placement to the last follow‐up or until death from any cause.

Duodenal invasion was diagnosed based on endoscopic findings (when duodenal erosions, ulcers, or strictures were observed and considered to be related to PC), regardless of pathological confirmation. The amount of ascites was classified using the Japanese Classification of Gastric Carcinoma: [15] mild, ascites localized in only one area; moderate, ascites neither mild nor massive; and massive, ascites throughout the abdomen. In this study, we defined procedure time as the time between endoscope insertion and stent deployment. However, we also measured the time from biliary cannulation to stent deployment (ERCP‐guided biliary drainage group) and the time from needle puncture to stent deployment (EUS‐HGS group), which definitions were used in a recent randomized controlled trial comparing ERCP with EUS‐BD [16]. The fasting period was defined as the interval between the index procedure and oral intake resumption. Oral intake was generally resumed when the patient was afebrile and didn't experience worsening abdominal pain. The final decision was left to the treating physician's discretion. Follow‐up data was confirmed until November 30, 2024.

### 2.4 Statistical Analysis

Categorical variables are described as numbers with proportions and were compared using the *χ*2 test or Fisher's exact test as appropriate. Continuous variables are presented as medians with ranges and were compared using the Mann‐Whitney U test. TRBO and OS were estimated using the Kaplan‐Meier method and were compared using the log‐rank test. Statistical significance was set at *p* values < 0.05. All statistical analyses were carried out using the EZR software version 1.40 [17].

## 3 Results

### 3.1 Patient Characteristics

**FIGURE 1:**
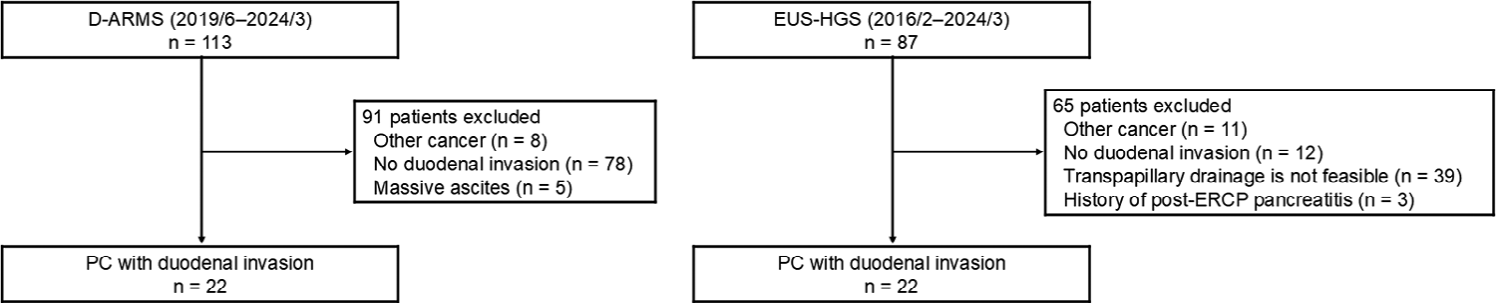
Patient flow chart.

Two hundred patients underwent ERCP‐guided biliary drainage with D‐ARMS (*n* = 113) or EUS‐HGS (*n* = 87) for MDBO during the study period. One hundred and fifty‐six patients were excluded and 44 patients were finally analyzed (D‐ARMS: 22; EUS‐HGS: 22) (Figure 1).

The D‐ARMS group had a significantly worse performance status and was less likely to receive chemotherapy after stent placement (59.1% vs. 95.2%, *p* = 0.009). Prior history of biliary drainage with SEMS (63.6% vs. 40.9%, *p* = 0.23) and prior biliary drainage with nasobiliary tube just before the index procedure (90.9% vs. 100%, *p* = 0.49) were similar between groups. No other patient characteristics significantly differed between groups including the location of duodenal invasion and the presence of co‐existing duodenal metal stent (31.8% vs. 13.6%, *p* = 0.28) (Table 1).

**TABLE 1:** Baseline characteristics.

	D‐ARMS *n* = 22	EUS‐HGS *n* = 22	*p*‐Value
Age, years	69 (46–79)	70 (46–78)	0.68
Sex, male	8 (36.4)	8 (36.4)	>0.99
Performance status, 0–1	17 (77.3)	22 (100)	0.049
Tumor status			>0.99
Locally advanced	6 (27.3)	6 (27.3)	
Metastatic or recurrent	16 (72.7)	16 (72.7)	
History of cholecystectomy	2 (9.1)	2 (9.1)	>0.99
Tumor involvement of orifice of cystic duct^a^	1 (5.0)	3 (15.0)	0.61
Tumor involvement of the pancreatic duct	19 (86.4)	20 (90.9)	>0.99
Ascites, moderate amount	3 (13.6)	1 (4.5)	0.61
Peritoneal dissemination	2 (9.1)	3 (13.6)	>0.99
Location of duodenal invasion			>0.99
Proximal/ampulla/distal	12 (54.5)/4 (18.2)/6 (27.3)	13 (59.1)/4 (18.2)/5 (22.7)	
Co‐existing duodenal metal stent	7 (31.8)	3 (13.6)	0.28
Prior history of biliary drainage with SEMS	14 (63.6)	9 (40.9)	0.23
Prior biliary drainage with ENBD just before D‐ARMS or EUS‐HGS	20 (90.9)	22 (100)	0.49
Chemotherapy after stent placement^b^	13 (59.1)	20 (95.2)	0.009

*Note*: Continuous variables are expressed as median (range) and categorical variables are expressed as absolute numbers (%).

Abbreviations: D‐ARMS, duckbill‐type anti‐reflux metal stent; ENBD, endoscopic nasobiliary biliary drainage; EUS‐HGS, endoscopic ultrasound‐guided hepaticogastrostomy; SEMS, self‐expandable metal stent.

^a^ Denominators adjusted to exclude four patients who underwent cholecystectomy (two patients in each group).

^b^ Denominators adjusted to exclude one patient in the EUS‐HGS group, where data on chemotherapy was missing because the patient was transferred to another hospital.

As the majority of patients received prior biliary drainage, the median procedure time was significantly shorter in the D‐ARMS group (16 vs. 50 min, *p* < 0.001). For EUS‐HGS, the median target branch diameter was 2.7 mm and the median time from needle puncture to stent deployment was 29 minutes. Insertion of a SEMS was unsuccessful in two patients in the EUS‐HGS group, and a 7Fr plastic stent was finally deployed in these cases. The SEMS used in each group were 10 mm in diameter with 6–8 cm in length in the D‐ARMS group and 8 mm in diameter with 10–12 cm in length in the EUS‐HGS group (Table 2).

**TABLE 2:** Procedural characteristics.

	D‐ARMS *n* = 22	EUS‐HGS *n* = 22	*p‐*Value
Procedure time, minutes	16 (8–66)	50 (13–130)	<0.001
Biliary cannulation to stent deployment	9 (5–25)		
Needle puncture to stent deployment		29 (12–122)	
Target branch, B2/B3		2 (9.1)/20 (90.9)	‐
Target branch diameter, mm		2.7 (1.2–4.0)	‐
Stent type			0.49
SEMS	22 (100)	20 (90.9)	
Plastic stent	‐	2 (9.1)	
Type of SEMS^a^			‐
Niti‐S S‐type Stent		4 (20.0)	
EGIS Biliary Stent, Double Covered		1 (5.0)	
Niti‐S Spring Stopper Stent		15 (75.0)	
D‐ARMS	22 (100)		
Stent diameter of SEMS^a^, mm			‐
8/10	0 (0)/22 (100)	20 (100)/0 (0)	
Stent length of SEMS^a^, cm			‐
6/7/8	10 (45.5)/1 (4.5)/11 (50.0)	‐	
10/12	‐	18 (90.0)/2 (10.0)	
Prophylactic pancreatic stent	1 (4.5)	0 (0)	‐
Prophylactic rectal NSAIDs use	8 (36.4)	0 (0)	‐

*Note*: Continuous variables are expressed as median (range) and categorical variables are expressed as absolute numbers (%).

Abbreviations: D‐ARMS, duckbill‐type anti‐reflux metal stent; EUS‐HGS, endoscopic ultrasound‐guided hepaticogastrostomy; NSAIDs, nonsteroidal anti‐inflammatory drugs; SEMS, self‐expandable metal stent.

^a^ Denominators adjusted to exclude two patients who received a plastic stent in the EUS‐HGS group.

**FIGURE 2:**
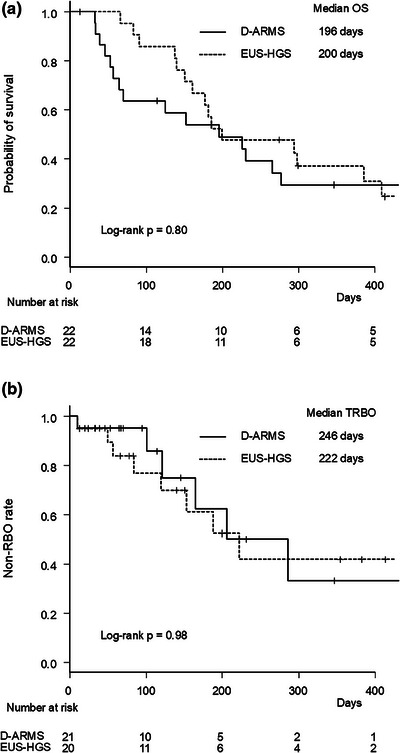
Kaplan‐Meier survival curves by stent group. (a) Overall survival. (b) Time to recurrent biliary obstruction. D‐ARMS, duckbill‐type anti‐reflux metal stent; EUS‐HGS, endoscopic ultrasound‐guided hepaticogastrostomy; OS, overall survival; RBO, recurrent biliary obstruction; TRBO, time to recurrent biliary obstruction.

Median OS was comparable between groups (196 vs. 200 days, *p* = 0.80) (Figure 2a).

### 3.2 Clinical Outcomes of ERCP‐guided Biliary Drainage and EUS‐HGS

The technical and clinical success rates were comparable between groups (Table 3). Two patients in the EUS‐HGS group who failed SEMS placement were considered a technical failure. One patient in the D‐ARMS group who underwent SEMS removal due to pancreatitis was considered a clinical failure, and the patient underwent EUS‐HGS on a different day. The fasting period was significantly shorter in the D‐ARMS group (1 vs. 3 days, *p* = 0.003). The incidence of overall AEs (RBO and non‐RBO AEs) was lower in the D‐ARMS group (36.4% vs. 63.6%, *p* = 0.13), although the difference was not statistically significant.

**TABLE 3:** Outcomes of endoscopic biliary drainage.

	D‐ARMS *n* = 22	EUS‐HGS *n* = 22	*p*‐Value
Technical success	22 (100)	20 (90.9)	0.49
Clinical success	21 (95.5)	20 (90.9)	>0.99
Fasting period, days	1 (1–6)	3 (1–6)	0.003
AEs	8 (36.4%)	14 (63.6)	0.13
Non‐RBO AEs	2 (9.1)	8 (36.4)	0.069
Early non‐RBO AEs^a^	2 (9.1)	4 (18.2)	0.66
Pancreatitis	2 (9.1)	‐	
Mild/moderate/severe	0/1/1		
Non‐occlusion cholangitis	0 (0)	1 (4.5)	
Mild/moderate/severe		1/0/0	
Bleeding	0 (0)	1 (4.5)	
Mild/moderate/severe		0/0/1	
Peritonitis	‐	2 (9.1)	
Mild/moderate/severe		0/1/1	
Late non‐RBO AEs^a^	0 (0)	5 (22.7)	0.049
Non‐occlusion cholangitis	0 (0)	3 (13.6)	
Mild/moderate/severe	‐	3/0/0	
Bleeding	0 (0)	1 (4.5)	
Mild/moderate/severe		0/0/1	
Liver abscess	0 (0)	1 (4.5)	
Mild/moderate/severe		0/0/1	
RBO^b^	7 (33.3)	9 (45.0)	0.53
Occlusion	3 (14.3)	9 (45.0)	
Biliary debris/stones	3	3	
Hyperplasia	0	4	
Hemorrhage	0	1	
Kinking	0	1	
Migration	2 (9.5)	0 (0)	
Complete distal migration	2		
Non‐occlusion cholangitis	2 (9.5)	0 (0)	

*Note*: Categorical variables are expressed as absolute numbers (%).

Abbreviations: AEs, adverse events; D‐ARMS, duckbill‐type anti‐reflux metal stent; EUS‐HGS, endoscopic ultrasound‐guided hepaticogastrostomy; RBO, recurrent biliary obstruction.

^a^ AEs were classified as early (within 14 days) and late (after 15 days) and their severity was graded according to the American Society of Gastrointestinal Endoscopy lexicon guidelines.

^b^ Denominators adjusted to exclude two patients who did not achieve technical success in the EUS‐HGS group and one patient who did not achieve clinical success in the D‐ARMS group.

The incidence of non‐RBO AEs was not significantly different between groups (9.1% vs. 36.4%, *p* = 0.069). Regarding early non‐RBO AEs, their incidence was similar between groups (9.1% vs. 18.2%, *p* = 0.66), albeit with different AE profiles. Pancreatitis developed in two patients in the D‐ARMS group. One patient removed the SEMS on the following day (the above‐mentioned case). The other patient developed pancreatitis 8 days after D‐ARMS placement and was initially treated with conservative treatment followed by EUS‐guided pseudocyst drainage. Peritonitis, bleeding, and non‐occlusion cholangitis occurred in two, one, and one patient, respectively, in the EUS‐HGS group, and all four patients were successfully treated conservatively. Regarding late non‐RBO AEs, their incidence was significantly higher in the EUS‐HGS group (0% vs. 22.7%, *p* = 0.049). Non‐occlusion cholangitis, bleeding, and liver abscess occurred in three, one, and one patient, respectively, in the EUS‐HGS group. Three patients were successfully treated conservatively, but one patient required transcatheter arterial embolization for late‐onset bleeding because of left hepatic artery pseudoaneurysm, which was related to the EUS‐HGS procedure.

The median follow‐up period was 114 days in the D‐ARMS group and 183 days in the EUS‐HGS group (*p* = 0.23). The RBO rates were similar between groups (33.3% vs. 45.0%, *p* = 0.53). The most common cause of RBO was biliary debris/stones in the D‐ARMS group and biliary debris/stones and hyperplasia in the EUS‐HGS group. Median TRBO was comparable between groups (246 vs. 222 days, *p* = 0.98). The non‐RBO rates at 3, 6, and 12 months were 95%, 63%, and 33%, respectively, in the D‐ARMS group, and 77%, 61%, and 42%, respectively, in the EUS‐HGS group (Figure 2b).

### 3.3 ERI after RBO

Technical and clinical success rates of ERI were 100% in both groups (Table 4). In the D‐ARMS group, three patients presented with gastric outlet obstruction (one patient with a co‐existing duodenal stent) and underwent simultaneous duodenal stenting and EUS‐BD. Two patients underwent EUS‐guided choledochoduodenostomy using D‐ARMS [7, 18] and one underwent EUS‐HGS. Of the remaining four patients, three underwent D‐ARMS placement after successful stent removal or stent migration and one underwent plastic stent placement in a stent‐in‐stent method. In the EUS‐HGS group, ERI was successfully performed through the EUS‐guided created route in all patients. Six patients underwent plastic stent placement in a stent‐in‐stent method and three were treated with balloon cleaning. The incidence of RBO rates after ERI was not significantly different between groups (0% vs. 22.2%, *p* = 0.48).

**TABLE 4:** Outcomes of endoscopic reintervention after first recurrent biliary obstruction.

	D‐ARMS *n* = 7	EUS‐HGS *n* = 9	*p*‐Value
Technical success	7 (100)	9 (100)	>0.99
Clinical success	7 (100)	9 (100)	>0.99
Details of reintervention			
EUS‐HGS	1 (14.3)		
EUS‐guided choledochoduodenostomy	2 (28.6)		
D‐ARMS replacement	3 (42.8)		
Stent‐in‐stent plastic stent placement	1 (14.3)	6 (66.7)	
Balloon cleaning		3 (33.3)	
RBO after reintervention	0 (0)	2 (22.2)	0.48

*Note*: Categorical variables are expressed as absolute numbers (%).

Abbreviations: D‐ARMS, duckbill‐type anti‐reflux metal stent; EUS‐HGS, endoscopic ultrasound‐guided hepaticogastrostomy; RBO, recurrent biliary obstruction.

## 4 Discussion

In this study, we examined the efficacy and safety of ERCP‐guided biliary drainage using D‐ARMS in comparison with EUS‐HGS in unresectable PC with duodenal invasion, where both procedures were technically feasible. While RBO rates and TRBO were comparable between groups, the procedure time and fasting period were significantly shorter in the D‐ARMS group, where prior biliary drainage was performed in most patients. Outcomes of ERI after the first RBO were comparable between groups, with three patients in the D‐ARMS group requiring conversion to EUS‐BD due to duodenal obstruction.

Duodenal invasion is considered a risk factor for early RBO because of the increased risk of duodenobiliary reflux [4, 5], indicating the need for new treatment strategies. The usage of a dedicated SEMS (D‐ARMS) and the creation of a bilio‐gastric anastomosis apart from the duodenum (EUS‐HGS) are two different strategies that may reduce the risk of duodenobiliary reflux and prevent early SEMS dysfunction. However, as no studies have compared these two strategies in patients with DMBO with duodenal invasion, it remains uncertain whether either procedure can provide better clinical outcomes.

A recent retrospective study [11] comparing EUS‐HGS with transpapillary drainage using conventional SEMS in PC patients with asymptomatic duodenal invasion showed that although median TRBO was comparable between groups (5.7 months [EUS‐HGS] vs. 8.8 months [conventional SEMS], *p* = 0.60), EUS‐HGS was associated with a lower rate of early RBO (≤ 3 months) (8% vs. 29%, *p* = 0.09). In this study, although median TRBO was comparable between groups (222 vs. 246 days, *p* = 0.89), the early RBO (≤ 3 months) rate was higher in the EUS‐HGS group compared to the D‐ARMS group (22.7% vs. 4.8%, *p* = 0.19). In addition, non‐RBO rates at 3 months were 52% in the EUS‐HGS group and 39% in the conventional SEMS group in the previous study [11], while the respective rates were 77% in the EUS‐HGS group and 95% in the D‐ARMS group in this study. Differences in outcomes between studies may be attributable to differences in patient characteristics and type of SEMS used in the ERCP‐guided biliary drainage group. In this study, we included cases with symptomatic duodenal invasion, with a co‐existing duodenal metal stent. The rate of co‐existing duodenal metal stent was higher in the D‐ARMS group (31.8% vs. 13.6%, *p* = 0.28), which might work to the disadvantage for the D‐ARMS group. Performance status and receipt of chemotherapy after stent placement were different between groups, albeit with comparable median OS. Even with such potential disadvantages in the D‐ARMS group, D‐ARMS showed similar efficacy compared to EUS‐HGS. We believe that the anti‐reflux valve of D‐ARMS was effective in preventing duodenobiliary reflux, resulting in a lower rate of early SEMS dysfunction compared to the conventional SEMS used in the previous study [11].

Consistent with the results of previous systematic reviews [19, 20, 21], early non‐RBO AE rates were comparable between groups (9.1% vs. 18.2%, *p* = 0.66), albeit with different safety profiles. Pancreatitis was a major concern for ERCP‐guided biliary drainage, while peritonitis and bleeding for EUS‐HGS. In this study, as we have placed a nasobiliary tube before the procedure in most patients, the incidence of pancreatitis and peritonitis might have been reduced compared to that when performed as the initial biliary drainage. On the other hand, late non‐RBO AE rates were significantly higher in the EUS‐HGS group (0% vs. 22.7%, *p* = 0.049). While most AEs were successfully treated conservatively, one patient required transcatheter arterial embolization for late‐onset bleeding, which may raise an alert for this procedure.

Several issues need to be discussed. While EUS‐HGS can be performed even when transpapillary biliary drainage is not feasible (e.g., patients with duodenal obstruction, surgically altered anatomy, or at high risk for post‐ERCP pancreatitis), it may be difficult to perform in some situations (e.g. patients with severe ascites, coagulopathy, or without bile duct dilatation). EUS‐HGS has not yet been widespread and recent guidelines still recommend this procedure at high‐volume expert centers [22]. RBO due to hyperplasia remains an issue to be resolved. A smaller diameter fully covered SEMS with an anti‐migration system may further improve the TRBO of EUS‐HGS [23]. In contrast, ERCP‐guided biliary drainage using D‐ARMS can be easily performed even in the above‐mentioned situations contraindicated for EUS‐HGS, when transpapillary biliary drainage is technically feasible. Although pancreatitis remains a concern, ERCP‐guided biliary drainage is more widespread than EUS‐HGS in the real‐world clinical setting.

This study has some limitations. First, this was a single‐center retrospective study with a limited sample size. Although both procedures were considered to be technically feasible in both groups, selection bias cannot be avoided. Performance status and receipt of chemotherapy after stent placement were different between groups, which might have affected the results of this study. This was partly because we expanded our indications for EUS‐HGS and performed EUS‐HGS at an earlier time point. Furthermore, some of the insignificant results of this study might have been due to the limited sample size. Second, most patients in both groups received prior biliary drainage with a nasobiliary tube before stent placement. Although this ensures that transpapillary biliary drainage was also technically feasible for those in the EUS‐HGS group, it remains uncertain whether the results of this study can be applied to those receiving initial biliary drainage.

In conclusion, RBO rates and TRBO were comparable between the two procedures. ERCP‐guided biliary drainage using D‐ARMS may be a viable option in the management of MDBO with duodenal invasion, especially for non‐high‐volume centers, when both procedures are technically feasible.

## Ethics Statement

**Approval of the research protocol by an Institutional Reviewer Board**: This study was approved by the institutional review board of our institution (2023‐GB‐077).

## Consent

Informed consent for this study was waived by the institutional review board owing to the retrospective study design.

## Conflicts of Interest

The authors declare no conflict of interest for this article. Conflicts of interest outside this work are as follows: Tsuyoshi Takeda received honoraria from Boston Scientific Japan and SB‐Kawasumi Laboratories; Takashi Sasaki received honoraria from Boston Scientific Japan, Century Medical, SB‐Kawasumi Laboratories, JAPAN LIFELINE, and KANEKA MEDIX; Naoki Sasahira received consulting fees from Gadelius Medical and honoraria from Olympus Medical and KANEKA MEDIX.

## Clinical Trial Registration

N/A.
